# Municipal distribution of bladder cancer mortality in Spain: Possible role of mining and industry

**DOI:** 10.1186/1471-2458-6-17

**Published:** 2006-01-27

**Authors:** Gonzalo Lopez-Abente, Nuria Aragones, Rebeca Ramis, Valentin Hernandez-Barrera, Beatriz Perez-Gomez, Antonio Escolar-Pujolar, Marina Pollan

**Affiliations:** 1 Environmental and Cancer Epidemiology Unit, National Centre for Epidemiology, Carlos III Institute of Health, Madrid, Spain; 2 Servicio de Medicina Preventiva, Hospital Universitario Puerta del Mar, Cádiz, Spain

## Abstract

**Background:**

Spain shows the highest bladder cancer incidence rates in men among European countries. The most important risk factors are tobacco smoking and occupational exposure to a range of different chemical substances, such as aromatic amines.

**Methods:**

This paper describes the municipal distribution of bladder cancer mortality and attempts to "adjust" this spatial pattern for the prevalence of smokers, using the autoregressive spatial model proposed by Besag, York and Molliè, with relative risk of lung cancer mortality as a surrogate.

**Results:**

It has been possible to compile and ascertain the posterior distribution of relative risk for bladder cancer adjusted for lung cancer mortality, on the basis of a single Bayesian spatial model covering all of Spain's 8077 towns. Maps were plotted depicting smoothed relative risk (RR) estimates, and the distribution of the posterior probability of RR>1 by sex. Towns that registered the highest relative risks for both sexes were mostly located in the Provinces of Cadiz, Seville, Huelva, Barcelona and Almería. The highest-risk area in Barcelona Province corresponded to very specific municipal areas in the Bages district, e.g., Suría, Sallent, Balsareny, Manresa and Cardona.

**Conclusion:**

Mining/industrial pollution and the risk entailed in certain occupational exposures could in part be dictating the pattern of municipal bladder cancer mortality in Spain. Population exposure to arsenic is a matter that calls for attention. It would be of great interest if the relationship between the chemical quality of drinking water and the frequency of bladder cancer could be studied.

## Background

In a European context, Spain ranks high in terms of bladder cancer mortality (2^nd^) in men, yet in women it figures among the countries with the lowest rates. During 2002, there were 3492 deaths in men and 703 in women due to this tumour, with an age-adjusted mortality rate (European population) of 14.01 in men and 1.78 in women per 100,000 population. Bladder cancer accounted for 6% and 2% of cancer deaths in men in women, respectively. In Spain, it is the 5^th ^leading cause of cancer-related death in men and the 13^th ^in women, with a sex ratio of 4:1. It is estimated that Spain has an average of 14,400 new cases per annum. The most frequent histological type is transitional cell (93%), followed by squamous cell cancer (2%) and adenocarcinomas (1%). The high bladder cancer survival rate (75% at 5 years in men and 70% in women) [1] means that partial prevalence (cases diagnosed in the last 5 years) is very high, viz., close on 40,000. This figure is very similar to that for the most frequent tumours, such as colorectal cancer, and thus constitutes an important public health problem here in Spain.

Spatial analysis of health events (spatial epidemiology) is a discipline that, though still in the development phase, is already enjoying a space of its own in the field of health research [2,3]. Its ability to suggest and detect the possible sources of heterogeneity (generally of environmental origin) which determine the spatial patterns of incidence and mortality due to different diseases, imbues this tool with great interest in the sphere of epidemiology and public health. Its potential is, moreover, being reinforced by the ever increasing availability of geographically-indexed population mortality and incidence data, together with advances in computation techniques and Geographic Information Systems. These circumstances are favouring the analysis of the geographical distribution of health data with growing levels of disaggregation [3], an area that encompasses the so-called small-area studies.

The main advantages of such small-area studies are: a) better interpretability of results in comparison with larger-scale studies; b) less susceptibility to ecological biases; and c) enhanced ability to detect local effects linked to environmental problems, such as industrial pollution [4].

The most important risk factor in bladder cancer is tobacco use, with the risk increasing in proportion to the intensity of smoking habit [5]. This tumour is also associated with occupational exposure to a range of chemical substances, such as 2-naphtilamine, benzidine and 4-aminobiphenyl, used in, among others, textile mills (dyeing and printing), the production of aromatic amines, and the rubber and leather industries [6-8]. Other environmental risk factors have also been described, such as exposure to the chemical elements of drinking water, whether of natural origin or the result of industrial activities, e.g., arsenic [9-13], or alternatively, exposure to compounds derived from water treatment/disinfection (trihalomethanes) [14]. The presence of inorganic arsenic in some foods [15-19] and its relationship with bladder cancer and other tumours is also under study [11,13], though low level arsenic exposure is unlikely to explain a substantial excess risk of bladder cancer [20].

It is assumed that the municipal spatial bladder cancer distribution pattern is fundamentally dictated by the prevalence of smokers, at least in men. The approach used in this study is based on the following idea: if it were possible "to extract" the smoking-habit component from municipal maps (confounding effect), the resulting pattern would then reflect the contribution of the remaining risk factors.

This study sought: a) to display maps depicting the municipal distribution of bladder cancer mortality; and b) to obtain the spatial pattern "adjusted" for the prevalence of smokers. Accordingly, on the assumption that lung cancer mortality is directly linked to the prevalence of tobacco smokers, this variable was used as a surrogate of tobacco use.

## Methods

As case source, we used individual death entries for the period 1989–1998 corresponding to bladder cancer (ICD-9 code 188) and lung cancer (ICD-9 code 162), broken down by town or city, nationwide. These data were furnished by the National Statistics Institute (*Instituto Nacional de Estadística *– *INE*) for the production of a municipal cancer mortality atlas, of which these results form part.

Municipal populations, broken down by age group (18 groups) and sex, were obtained from the 1991 census and 1996 municipal roll. These years correspond to the midpoints of the two quinquennia that comprise the study period (1989–1993 and 1994–1998). The person-years for each five-year period were obtained by multiplying these populations by 5.

Standardised mortality ratios (SMR) were calculated as the ratio of observed and expected deaths. For the calculation of expected cases, the overall Spanish mortality rates for the above two 5-year periods were multiplied by each town's person-years by age group, sex and quinquennium.

For map plotting purposes, smoothed municipal relative risks (RR) were calculated using the conditional autoregressive model proposed by Besag, York and Molliè (BYM). This model was introduced by Clayton and Kaldor [21], developed by Besag, York and Molliè [22], and subsequently applied in the field of ecological studies [23]. These models are based on fitting Poisson spatial models with observed cases as the dependent variable, expected cases as offset, and two types of random effects terms which take the following into account: a) municipal contiguity (spatial term); and b) municipal heterogeneity.

The models were fitted using Bayesian Markov chain Monte Carlo simulation methods with non-informative priors [24]. Posterior distributions of relative risk were obtained using WinBugs [25]. The criterion of contiguity used was adjacency of municipal boundaries. Convergence of the simulations was verified using the BOA (Bayesian Output Analysis) R program library [26]. Given the great number of parameters of the models, the convergence analysis was performed on a randomly selected sample of 10 towns and cities, taking 4 strata defined by municipal size. Convergence of the estimators was achieved before 100,000 iterations. For the maps shown, a "burn-in" (iterations discarded to ensure convergence) of 300,000 iterations was performed and the posterior distribution was derived with 5,000.

Thereafter a model was constructed for bladder cancer, similar to the above model but adding, as another fixed-effects term, the posterior distribution of RR for lung cancer normalised by means of its logarithm.

A Geographic Information System was used to plot municipal maps that depicted smoothed RR estimates and the distribution of the posterior probability (pp) that RR>1 (Bayesian version of p value). With regard to this indicator, we followed Richardson's criterion [4], which recommends that probabilities above 0.8 should be deemed significant.

Separate analyses were performed for men and women.

## Results

From 1989 to 1998, a total of 34281 bladder cancer deaths were registered in Spain, 28173 in men and 6108 in women. In 3824 towns and cities no death due to this cause was registered. Using these data, an acceptable computation time and conventional computers, it was possible to compile and ascertain the posterior distribution of relative risk on the basis of a single spatial model that included all of Spain's 8077 towns and cities and the 46398 adjacencies existing between them. Table 1 displays a number of descriptive statistics for the population and disease data. The total population was just under 40 million, and lung cancer mortality was four times higher than that of bladder cancer, with the mean number of cases per area being 19 and 4 for lung and bladder cancer, respectively.

**Table 1:** Summaries of population and cancer mortality in the 8077 Spanish towns. Spain 1989–1998.

	Total	Mean	Median	Standard deviation	Minimum	Maximum	No. (%) of areas with zero counts
Population	39648759	4908.85	586	42430.43	5	2866850	0 (0)
Observed bladder	34281	4.24	1	40.77	0	2543	3823 (47.3)
Expected bladder	34333	4.25	0.92	39.33	0.01	2721.16	0 (0)
Bladder SMRs^1^	-	0.76	0.39	1.19	0	26.32	3823 (47)
Observed lung	155014	19.19	3	190.03	0	12434	1905 (24.6)
Expected lung	155207	19.22	3.79	177.00	0.04	12243.54	0 (0)
Lung SMRs^1^	-	0.72	0.69	0.64	0	14.82	1905 (24.6)

^1^Descriptive values of the 8077 SMRs

**Figure 1:**
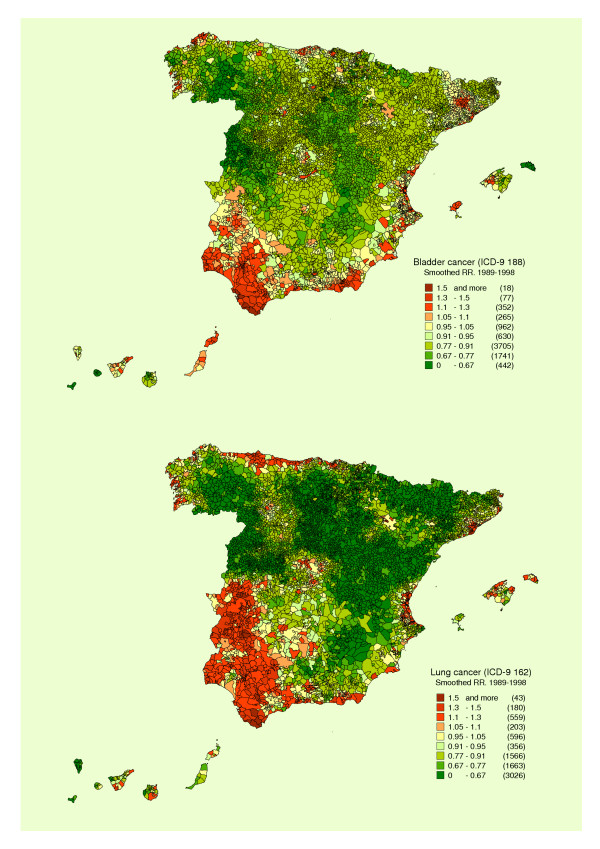
Municipal distribution of bladder and lung cancer mortality in Spain. Distribution pattern of smoothed relative risk (RR) under the BYM model. Spain 1989–1998.

Figure 1 depicts the distribution of the smoothed RR: a) for bladder cancer; and b) for lung cancer (both sexes). The patterns of both tumours display similarities as well as differences: the similarities consist of the higher risk found in many of the towns and cities in the Provinces of Cadiz, Huelva and Seville, in Almería, and along the coast of the Valencian Region; the most marked differences correspond to Extremadura, Asturias, and Barcelona Province.

**Figure 2:**
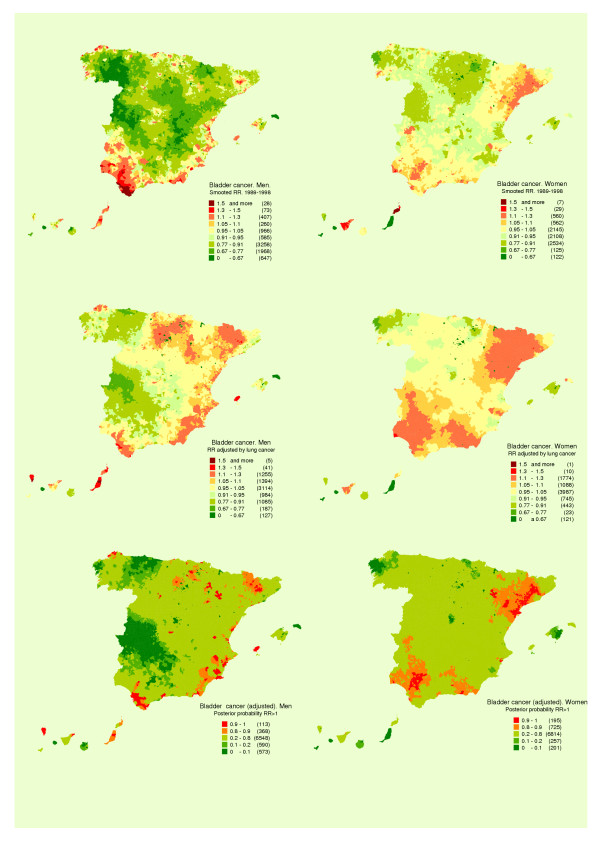
Municipal distribution of bladder cancer mortality: a) men, b) women, c) and d) men and woman respectively adjusted for lung cancer mortality, e) and f) posterior probability of RR being greater than 1. Spain 1989–1998.

Figures 2a and 2b show the distributions of bladder cancer in men and women. The patterns were different, with less variability in women, yet in both sexes there were 3 large areas, namely, Western Andalusia, Almería and Catalonia.

Shown in Figures 2c and 2d is the distribution of RR for bladder cancer "adjusted" for distribution of the log(RR) in lung cancer. This map highlights the differences between the bladder and lung cancer maps. Figures 2e and 2f "filter" the previous figures, flagging the areas in which excess mortality is more likely.

The towns and cities that registered a risk in excess of 1.30 (Figures 2a and 2b), totalled 101 in men and 36 in women, and belonged, for the most part to the Provinces of Cadiz, Seville, Huelva, Barcelona and Almería. Table 2 shows the results for towns and cities that, with at least 5 observed cases, registered adjusted RR of 1.3 or more and pp ≥ 0.9 in men or women.

**Table 2:** Bladder cancer mortality in Spain. Towns with 5 or more observed cases, which have shown an RR adjusted for lung cancer of over 1.3 in men or women, with a posterior probability (pp) of over 0.9 that RR is greater than 1 (p(RR>1)). Towns listed in order of province. Spain 1989–1998.

Town		Both sexes						Men				Women			
NAME	INE	Obs	Exp	SMR	RR	pp	RR adj	RR	pp	RR adj	pp adj	RR	pp	RR adj	pp adj

EIVISSA	7026	31	17.87	1.74	1.49	0.99	1.38	1.64	1.00	1.50	1.00	0.81	0.30	0.79	0.23
SAN JOSE	7048	9	7.55	1.19	1.26	0.83	1.30	1.29	0.84	1.38	0.95	0.86	0.34	0.82	0.25
STA EULALIA DEL RIO	7054	16	13.18	1.21	1.23	0.85	1.33	1.26	0.87	1.39	0.96	0.86	0.33	0.81	0.25
BALSARENY	8018	9	3.82	2.35	1.41	0.96	1.38	1.48	0.96	1.38	0.98	1.13	0.76	1.23	0.89
CARDONA	8047	19	7.41	2.57	1.40	0.97	1.43	1.38	0.97	1.40	0.99	1.14	0.77	1.21	0.93
MANRESA	8113	93	70.84	1.31	1.24	0.99	1.24	1.11	0.82	1.12	0.88	1.42	0.99	1.34	0.99
MATARO	8121	109	77.07	1.41	1.32	1.00	1.17	1.25	0.98	1.08	0.77	1.36	0.98	1.32	0.97
SALLENT	8191	20	9.24	2.16	1.45	0.99	1.42	1.33	0.95	1.33	0.98	1.28	0.92	1.28	0.97
SURIA	8274	16	6.70	2.39	1.53	0.99	1.45	1.49	0.97	1.39	0.98	1.22	0.84	1.28	0.94
BURGOS	9059	168	134.86	1.25	1.17	0.98	1.33	1.21	0.99	1.37	1.00	0.93	0.25	1.01	0.49
ALGECIRAS	11004	109	62.39	1.75	1.71	1.00	1.25	1.89	1.00	1.33	1.00	1.00	0.51	0.99	0.45
CADIZ	11012	177	97.70	1.81	1.80	1.00	1.40	1.86	1.00	1.39	1.00	1.46	0.99	1.51	0.99
PUERTO DE STA MARIA	11027	70	33.62	2.08	1.90	1.00	1.43	2.08	1.00	1.49	1.00	1.08	0.67	1.12	0.77
PUERTO REAL	11028	30	15.08	1.99	1.73	1.00	1.37	1.79	1.00	1.37	0.99	1.17	0.82	1.19	0.88
ROTA	11030	27	12.55	2.15	1.83	1.00	1.38	1.97	1.00	1.45	1.00	1.08	0.64	1.12	0.71
SAN FERNANDO	11031	74	45.04	1.64	1.63	1.00	1.39	1.60	1.00	1.34	1.00	1.42	0.97	1.39	0.98
NARON	15054	38	25.05	1.52	1.35	0.98	1.32	1.35	0.97	1.31	0.98	1.07	0.60	1.00	0.45
CARIÑO	15901	16	6.29	2.55	1.75	0.99	1.43	1.80	0.99	1.43	0.98	1.09	0.58	1.03	0.52
LEPE	21044	14	9.72	1.44	1.38	0.95	1.06	1.29	0.89	0.99	0.44	1.30	0.81	1.33	0.91
SOLSONA	25207	14	7.48	1.87	1.34	0.89	1.44	1.26	0.82	1.37	0.95	1.22	0.76	1.28	0.87
PUERTO DEL ROSARIO	35017	12	6.75	1.78	1.22	0.75	1.28	1.37	0.86	1.51	0.94	0.36	0.03	0.24	0.02
BREÑA BAJA	38009	5	3.20	1.56	1.04	0.51	1.41	1.05	0.53	1.52	0.97	0.79	0.26	0.82	0.23
LLANOS DE ARIDANE	38024	21	13.83	1.52	1.15	0.75	1.53	1.23	0.84	1.68	1.00	0.75	0.22	0.80	0.20
VILLA DE MAZO	38053	6	5.80	1.04	0.99	0.44	1.41	1.01	0.48	1.53	0.98	0.78	0.26	0.83	0.23
TOCINA	41092	13	6.08	2.14	1.52	0.97	1.10	1.38	0.91	1.00	0.48	1.25	0.80	1.33	0.92
ELIANA	46116	14	5.42	2.59	1.46	0.97	1.26	1.62	0.98	1.32	0.96	0.88	0.21	0.96	0.36

INE = municipal code; Obs Exp = observed and expected deaths; RR = estimated relative risk; SMR = standard mortality ratio. RR adj = estimate adjusted for lung cancer. pp = posterior probability that RR>1.

The highest risk area recorded by Barcelona Province corresponded to very specific municipal areas in the Bages district, e.g., Suría, Sallent, Balsareny, Manresa and Cardona. Other towns with relative risks in excess of 1.3 were situated in Murcia, Burgos and Corunna (*A Coruña*).

Towns in Cadiz, Seville and Huelva registered a certain excess risk for both sexes. In view of the peculiar spatial pattern of these towns, it would seem that there are determinants other than the high prevalence of smokers accounting for the bladder cancer mortality pattern in this area, since this same pattern is also applicable to women. In Almería a sizeable area of raised risk was in evidence.

Barcelona Province was one of the areas that deserve some comments. Two areas of higher risk were identified, one comprising the Bages district (Suria, Sallent, Balsareny, Manresa and Cardona), and the other flanking the coast (Vilanova i la Geltru, Mataró), Barcelona, Barbera, Moncada and Santa Coloma. In the Bages district, excess risk was detected in both sexes: in the case of women, however, not only were the Bages towns identified, but the areas marked on map were also larger. The posterior probability map identifies towns in Vallés, Maresme and Baix Llobregat and an area in Tarragona around the Delta of the River Ebro.

## Discussion

The municipal geographical bladder cancer pattern is similar to that plotted by lung cancer, possibly reflecting the role of tobacco smoking, the leading etiological agent. The difference in patterns with respect to lung cancer in terms of both geographical distribution and time trend [27] might be attributable to the existence of occupational exposures and environmental differentials. In addition, the differences with other countries in terms of mortality frequency and trend [28] may possibly be related to black tobacco (dark air cured tobacco) use among men, an exposure which, for this tumour, has shown a higher risk than that for other types of tobacco [6].

The origin of bladder cancer is determined by contact between the vesical epithelium and carcinogenic substances excreted in urine. Such substances may be ingested or inhaled directly, or come from the metabolism of other products in the body. As mentioned above, the two risk factors acknowledged as being most important for this type of cancer are smoking and occupational exposure to aromatic amines. Among the occupations associated with a higher risk of bladder cancer are those linked to the production of aromatic amines, rubber manufacture, exposure to dyes and printing in the textile industry [7], paint, aluminium, tanning and curing of hides, and the driving of motor vehicles [6,8].

The risk associated with the consumption of chlorinated drinking water was evaluated in different occasions. A recent published metaanalysis indicates that long term consumption of chlorinated drinking water is associated with a 40% of increased risk of bladder cancer incidence, particularly in men [14,29]. Those studies found an increased risk of bladder cancer and a dose-response pattern among men exposed to trihalomethanes at levels currently observed in many industrialized countries. However they did not find an increased risk in women and this observed difference in risk by sex is puzzling.

The municipal mortality pattern shown in Spain needs to include another elements among possible explanations. In the recent years, the role of other environmental exposures, for instance arsenic, in the aetiology of bladder cancer (and other tumours), has gradually become recognised. Inorganic arsenic has been documented as being a skin and lung carcinogen [30,31]. A number of studies have observed a rise in bladder and kidney cancer mortality in populations with high exposure to arsenic in drinking water [9,10,32,33] and, in the case of bladder cancer, a dose-response relationship has been reported [11,12].

The method applied to smooth geographical patterns, with the limitations commented below, is currently the most widely accepted method for data studies focusing on small areas [2,3]. One aspect that merits comment is the use of lung cancer mortality as a surrogate variable to adjust for exposure to tobacco. A single aetiological agent, cigarette smoking, is by far the leading cause of lung cancer, accounting for approximately 90% of lung cancer cases in countries where cigarette smoking is common [34]. Nowadays, the risk of lung cancer posed to smokers is about 20 times greater than that posed to never-smokers [34]. Few exposures to environmental agents convey such risks for any disease. The unequivocal causal association between cigarette smoking and lung cancer is one of the most thoroughly documented causal relationships in biomedical research [34]. Hence, to consider the distribution of lung cancer as a good indicator of the prevalence of smokers is by no means illogical, although this ecological approach is subject to residual confounding.

The joint modelling of several diseases is something that has been addressed on different occasions with varying approaches [35-37]. Recently, two papers have been published on spatial variation in the rates of several diseases with common risk factors, an approach known as "joint disease mapping' [38,39]. Our method of analysis is described as an 'ecological regression' in the paper by Held et al., and in their paper, Dabney et al. provide examples of modelling procedures with lung and bladder cancer incidence.

In our results, the effect of adjusting for lung cancer is to produce a "filtering" of the geographical pattern, extracting from the maps those towns and cities in which bladder cancer mortality can be explained by tobacco use (Figure 2c and 2d).

Most of the municipal areas identified by the models as representing a raised risk for both sexes are situated in the Provinces of Cadiz, Seville and Huelva. On the map adjusted for lung cancer, the towns that are highlighted are listed in order of their proximity to the River Guadalquivir. This, coupled with the fact that this pattern is also seen among women, is indicative that there are environmental components other than the prevalence of smokers underlying bladder cancer mortality in this area. The environs of the Huelva estuary and the Campo de Gibraltar district have been the subject of study and specific reports by the Scientific Research Board (*Consejo Superior de Investigaciones Científicas *– *CSIC*) on the serious situation of their environmental pollution, with high concentrations of heavy metals being detected in sediments throughout the area [40].

The content of these reports [40] and the municipal mortality patterns suggest that industrial activity in the Province of Cadiz, and industrial and mining activity in the Provinces of Seville and Huelva could be associated with bladder cancer mortality in these provinces. Furthermore, bearing in mind the levels of arsenic and other heavy metals in fish and shellfish [15,41,42], questions might well be raised about the possible role played in the incidence of this tumour by the high consumption of fish in Spain [43] and regional differences in such consumption. The maximum limits of arsenic in foodstuffs are still not regulated [40]. In comparison with other European countries, however, Spain has high rates in men only, with bladder cancer in women being extremely infrequent. On the other hand, however, risk of bladder cancer for exposure to low doses of arsenic is reported to have been observed only in smokers [44,45].

In the smoothed maps for Barcelona Province, two areas with higher than expected mortality were detected, one in the Bages district and the other in Vallés, Maresme and Baix Llobregat. These two areas differ, in that whereas in the former the risk is higher in both sexes, in the latter this is true only of women (Figures 2a and 2b).

Wholly different explanations might account for these patterns. The Bages pattern affects men and women, which could be interpreted as due to some environmental exposure other than occupation. The Bages district has been characterised by sodium and potash mining and the textile industry. The mining industry faithfully reflects the towns of Cardona (salt mines worked from 1923 to 1990 by ERCROS), Suria, Sallent, Balsareny (sodium chloride and potash mines, run by Iberpotash). Mining-specifically salt mining [8] – is one of the occupations in which an association has been described with bladder cancer. High concentrations of arsenic have been detected in the Cardener and Llobregat Rivers, concentrations that have clearly increased due to the influence of the mining activity in Sallent-Balsareny [46]. Moreover, in the 1930s the Bages district and Llobregat basin were the leading cotton-based textile industry areas in Catalonia [47].

The Vallés/Maresme pattern, which is only observable in women, might be related to the textile industry traditionally situated in these areas. Textiles, particularly cotton, constituted the strongest sector by far in Catalonian industrialisation, a sector characterised by an abundant presence of women workers. Dyeing and printing are the sections that have shown an association with bladder cancer in the literature [7]. Posterior studies conducted in Barcelona province do not support the findings respective to the textile industry although they found very high relative risks in workers employed for very long [48]. In a pooled analysis of occupation and bladder cancer in women, the increased risk in textile industry is for exposed for more than 25 years [49]. In a more recent update the opinion is that there is a general tendency to move toward unity for all the cancer relative risk estimates in textile industry workers [50]. Some exposures to carcinogens could be limited in time, with the improving of industrial procedures.

Some authors have described an association between arsenic levels in drinking water and the incidence of certain tumours, including transitional cell carcinomas of the urinary bladder [51]. It could be argued that maximum levels of arsenic in drinking water are limited by law and are routinely monitored, so that any substantial excess risk would be difficult to explain. Nevertheless, the limit on arsenic levels in water is currently under discussion. Until 1990 the maximum permissible level in Spain of arsenic intended for human consumption was 100 μg/l. This was then reduced to 50 μg/l. Subsequently, the European Council Directive 98/83/EC of 3 November 1998 on the quality of water intended for human consumption, applied the World Health Organisation (WHO) guideline and set the maximum admissible concentration for this element at 10 μg/l, the level adopted in Spain at the end of 2003 (Royal Decree 140/2003). These data not only attest to the controversy surrounding the health risk of ingesting inorganic arsenic and the advisability of present and future limits [52,53], but also mean that for many years the permitted levels of arsenic in drinking water have been up to 10 times higher than those now tolerated. The hypothesis of exposure to arsenic would be reinforced, if the proportion of the population that drinks well-water and is thus more difficult to control, were higher in areas with higher risks of bladder cancer, but this is something that we do not know.

The use of arsenic-based pesticides has been viewed as the principal source of arsenic-related environmental pollution in recent decades [17]. Phytosanitary products are used intensively in areas like Almería, a factor that calls for detailed study.

Arsenic can also been found in certain pesticides and in wood preservatives. In the past, arsenic was primarily used as a pesticide, primarily on cotton fields and in orchards. Inorganic arsenic compounds can no longer be used in agriculture. However, organic arsenicals, namely cacodylic acid, disodium methylarsenate (DSMA), and monosodium methylarsenate (MSMA) are still used as pesticides, principally on cotton [54].

Andalusia holds almost 95% of cotton cultivated surface, which represents 2% of its cropland. Cotton crops are mainly found in Occidental Andalusia (Seville, Cadiz, Cordoba), in the region known as " Bajo Guadalquivir", and to a much lesser extent, in Huelva. In "Bajo Guadalquivir" other usual crops are sunflower, beetroot and cereals such as rice, in which pesticide use has also been quite common.

Another possible source of arsenic might be occupational exposure to chromated copper arsenate (CCA), which is an inorganic arsenic compound commonly used as wood preservative for outdoor furnitures [55,56]. The marketing and use of this substance (CCA) as wood preservant and the commercialization of the products treated with it has been recently banned (COMMISSION DIRECTIVE 2003/2/ES january 6, 2003).

Geographical small-area studies such as ours, though targeted at generating aetiological hypotheses, can be efficient instruments for detecting problems connected with environmental quality and, in addition, furnish very flexible information which enables some of the limitations posed by classic ecological studies to be overcome. The results suggest that mining/industrial pollution and the risk entailed in certain occupational exposures could in part be dictating the pattern of municipal bladder cancer mortality in Spain. Population exposure to arsenic found in diet and drinking water is a matter that calls for attention. Accordingly, it would seem advisable to study the possible restriction of inorganic arsenic levels in foods and to explore whether some relationship exists between the chemical quality of drinking water and the frequency of bladder cancer in Spain. Also the use of arsenical pesticides deserve attention in Occidental Andalucía and Almería.

## Competing interests

The author(s) declare that they have no competing interests.

## Authors' contributions

GLA was responsible for the development of intellectual content and the study design, interpretation of the results and manuscript drafting. MP, NA, BPG contributed with the study design, the improving of mortality modelling and the critical revision of manuscript. AE contributed in the critical review of results and discussion. RR, VH performed part of the statistical analysis and contributed to manuscript drafting. All authors read and approved the final manuscript.

## Pre-publication history

The pre-publication history for this paper can be accessed here:



## References

[B1] Coleman MP, Gatta G, Verdecchia A, Esteve J, Sant M, Storm H, Allemani C, Ciccolallo L, Santaquilani M, Berrino F (2003). EUROCARE-3 summary: cancer survival in Europe at the end of the 20th century. Ann Oncol.

[B2] Lawson A, Biggeri A, Bohning D, Lesaffre E, Biggeri A, Viel JF (1999). Disease mapping and risk assessment for public health.

[B3] Elliott P, Wakefield JC, Best N, Briggs S (2000). Spatial epidemiology.

[B4] Richardson S, Thomson A, Best N, Elliott P (2004). Interpreting posterior relative risk estimates in disease-mapping studies. Environ Health Perspect.

[B5] Lopez-Abente G, Gonzalez CA, Errezola M, Escolar A, Izarzugaza I, Nebot M, Riboli E (1991). Tobacco smoke inhalation pattern, tobacco type, and bladder cancer in Spain. Am J Epidemiol.

[B6] Silverman DT, Hartge P, Morrison AS, Devesa SS (1992). Epidemiology of bladder cancer. Hematol Oncol Clin North Am.

[B7] Gonzalez CA, Lopez-Abente G, Errezola M, Escolar A, Riboli E, Izarzugaza I, Nebot M (1989). Occupation and bladder cancer in Spain: a multi-centre case-control study. Int J Epidemiol.

[B8] Kogevinas M, Mannetje A., Cordier S, Ranft U, Gonzalez CA, Vineis P, Chang-Claude J, Lynge E, Wahrendorf J, Tzonou A, Jockel KH, Serra C, Porru S, Hours M, Greiser E, Boffetta P (2003). Occupation and bladder cancer among men in Western Europe. Cancer Causes Control.

[B9] Chen CJ, Chuang YC, You SL, Lin TM, Wu HY (1986). A retrospective study on malignant neoplasms of bladder, lung and liver in blackfoot disease endemic area in Taiwan. Br J Cancer.

[B10] Smith AH, Goycolea M, Haque R, Biggs ML (1998). Marked increase in bladder and lung cancer mortality in a region of Northern Chile due to arsenic in drinking water. Am J Epidemiol.

[B11] Chiou HY, Hsueh YM, Liaw KF, Horng SF, Chiang MH, Pu YS, Lin JS, Huang CH, Chen CJ (1995). Incidence of internal cancers and ingested inorganic arsenic: a seven-year follow-up study in Taiwan. Cancer Res.

[B12] Tsuda T, Babazono A, Yamamoto E, Kurumatani N, Mino Y, Ogawa T, Kishi Y, Aoyama H (1995). Ingested arsenic and internal cancer: a historical cohort study followed for 33 years. Am J Epidemiol.

[B13] Hopenhayn-Rich C, Biggs ML, Smith AH (1998). Lung and kidney cancer mortality associated with arsenic in drinking water in Cordoba, Argentina. Int J Epidemiol.

[B14] Villanueva CM, Cantor KP, Cordier S, Jaakkola JJ, King WD, Lynch CF, Porru S, Kogevinas M (2004). Disinfection byproducts and bladder cancer: a pooled analysis. Epidemiology.

[B15] Llobet JM, Falco G, Casas C, Teixido A, Domingo JL (2003). Concentrations of arsenic, cadmium, mercury, and lead in common foods and estimated daily intake by children, adolescents, adults, and seniors of Catalonia, Spain. J Agric Food Chem.

[B16] Bordajandi LR, Gomez G, Abad E, Rivera J, Mar Fernandez-Baston M, Blasco J, Gonzalez MJ (2004). Survey of persistent organochlorine contaminants (PCBs, PCDD/Fs, and PAHs), heavy metals (Cu, Cd, Zn, Pb, and Hg), and arsenic in food samples from Huelva (Spain): levels and health implications. J Agric Food Chem.

[B17] Urieta I, Jalon M, Eguilero I (1996). Food surveillance in the Basque Country (Spain). II. Estimation of the dietary intake of organochlorine pesticides, heavy metals, arsenic, aflatoxin M1, iron and zinc through the Total Diet Study, 1990/91. Food Addit Contam.

[B18] Delgado-Andrade C, Navarro M, Lopez H, Lopez MC (2003). Determination of total arsenic levels by hydride generation atomic absorption spectrometry in foods from south-east Spain: estimation of daily dietary intake. Food Addit Contam.

[B19] Herce-Pagliai C, Moreno I, Gonzalez G, Repetto M, Camean AM (2002). Determination of total arsenic, inorganic and organic arsenic species in wine. Food Addit Contam.

[B20] Michaud DS, Wright ME, Cantor KP, Taylor PR, Virtamo J, Albanes D (2004). Arsenic concentrations in prediagnostic toenails and the risk of bladder cancer in a cohort study of male smokers. Am J Epidemiol.

[B21] Clayton D, Kaldor J (1987). Empirical Bayes estimates of age-standardized relative risks for use in disease mapping. Biometrics.

[B22] Besag J, York J, Molliè A, Besag J, York JC, Mollie A (1991). Bayesian image restoration, with two applications in spatial statistics (with discussion).. Annals of the Institute of Statistical Mathematics.

[B23] Clayton DG, Bernardinelli L, Montomoli C (1993). Spatial correlation in ecological analysis. Int J Epidemiol.

[B24] Gilks W, Richardson S, Spiegelhalter D (1995). Markov Chain Monte Carlo in Practice Interdisciplinary Statistics.

[B25] Spiegelhalter D, Thomas A, Best N, Lunn D (2003). WinBUGS user manual Version 141.

[B26] Smith BJ (2001). Bayesian Output Analysis Program (BOA), Version 0.99.1 for S-PLUS and R. http://www.public-health.uiowa.edu/BOA.

[B27] Lopez-Abente G, Pollán M, Aragones N, Pérez B, Llácer A, Pérez J (2002). Tendencias de la mortalidad en España, 1952–1996 Efecto de la edad, de la cohorte de nacimiento y del periodo de muerte.

[B28] Ferlay J, Bray F, Sankila R, Parkin D (1999). EUCAN: Cancer Incidence, Mortality and Prevalence in the European Union 1998, version 50.

[B29] Villanueva CM, Fernandez F, Malats N, Grimalt JO, Kogevinas M (2003). Meta-analysis of studies on individual consumption of chlorinated drinking water and bladder cancer. J Epidemiol Community Health.

[B30] IARC (International Agency for Research on Cancer) (1987). IARC monographs on the evaluation of carcinogenic risks to humans: overall evaluations of carcinogenicity An updating of IARC monographs volumes 1 to 42.

[B31] EPA (Environmental Protection Agency) (1988). Risk assessment forum Special report on ingested inorganic arsenic: skin cancer, nutrition essentiality.

[B32] Wu MM, Kuo TL, Hwang YH, Chen CJ (1989). Dose-response relation between arsenic concentration in well water and mortality from cancers and vascular diseases. Am J Epidemiol.

[B33] Hopenhayn-Rich C, Biggs ML, Fuchs A, Bergoglio R, Tello EE, Nicolli H, Smith AH (1996). Bladder cancer mortality associated with arsenic in drinking water in Argentina. Epidemiology.

[B34] Alberg AJ, Samet JM (2003). Epidemiology of lung cancer. Chest.

[B35] Langford IH, Leyland AH, Rasbash J, Goldstein H (1999). Multilevel modelling of the geographical distributions of diseases. J R Stat Soc Ser C Appl Stat.

[B36] Leyland AH, Langford IH, Rasbash J, Goldstein H (2000). Multivariate spatial models for event data. Stat Med.

[B37] Knorr-Held L, Best N (2001). A shared component model for detecting joint and selective clustering of two diseases. Journal of the Royal Statistical Society, Series A.

[B38] Held L, Natario I, Fenton SE, Rue H, Becker N (2005). Towards joint disease mapping. Stat Methods Med Res.

[B39] Dabney AR, Wakefield JC (2005). Issues in the mapping of two diseases. Stat Methods Med Res.

[B40] CSIC (Consejo Superior de Investigaciones Científicas) (2005). Informes del estudio sobre el diagnóstico ambiental y sanitario de la ría de Huelva. Segundo informe del estudio que coordina el Consejo Superior de Investigaciones Científicas sobre el diagnóstico ambiental y sanitario de la ría de Huelva. http://www.csic.es/wi/informes_csic.jsp.

[B41] Sanzo JM, Dorronsoro M, Amiano P, Amurrio A, Aguinagalde FX, Azpiri MA (2001). Estimation and validation of mercury intake associated with fish consumption in an EPIC cohort of Spain. Public Health Nutr.

[B42] Schuhmacher M, Batiste J, Bosque MA, Domingo JL, Corbella J (1994). Mercury concentrations in marine species from the coastal area of Tarragona Province, Spain. Dietary intake of mercury through fish and seafood consumption. Sci Total Environ.

[B43] Welch AA, Lund E, Amiano P, Dorronsoro M, Brustad M, Kumle M, Rodriguez M, Lasheras C, Janzon L, Jansson J, Luben R, Spencer EA, Overvad K, Tjonneland A, Clavel-Chapelon F, Linseisen J, Klipstein-Grobusch K, Benetou V, Zavitsanos X, Tumino R, Galasso R, Bueno-De-Mesquita HB, Ocke MC, Charrondiere UR, Slimani N (2002). Variability of fish consumption within the 10 European countries participating in the European Investigation into Cancer and Nutrition (EPIC) study. Public Health Nutr.

[B44] Karagas MR, Tosteson TD, Morris JS, Demidenko E, Mott LA, Heaney J, Schned A (2004). Incidence of transitional cell carcinoma of the bladder and arsenic exposure in New Hampshire. Cancer Causes Control.

[B45] Bates MN, Rey OA, Biggs ML, Hopenhayn C, Moore LE, Kalman D, Steinmaus C, Smith AH (2004). Case-control study of bladder cancer and exposure to arsenic in Argentina. Am J Epidemiol.

[B46] Rosas Rodríguez H (2001). Estudio de la contaminación por metales pesados en la cuenca del Llobregat Doctoral thesis.

[B47] Nadal J (1992). Moler, tejer y fundir Estudios de historia industrial.

[B48] Serra C, Bonfill X, Sunyer J, Urrutia G, Turuguet D, Bastus R, Roque M, 't MA, Kogevinas M (2000). Bladder cancer in the textile industry. Scand J Work Environ Health.

[B49] Mannetje A, Kogevinas M, Chang-Claude J, Cordier S, Gonzalez CA, Hours M, Jockel KH, Bolm-Audorff U, Lynge E, Porru S, Donato F, Ranft U, Serra C, Tzonou A, Vineis P, Wahrendorf J, Boffetta P (1999). Occupation and bladder cancer in European women. Cancer Causes Control.

[B50] Mastrangelo G, Fedeli U, Fadda E, Milan G, Lange JH (2002). Epidemiologic evidence of cancer risk in textile industry workers: a review and update. Toxicol Ind Health.

[B51] Luster MI, Simeonova PP (2004). Arsenic and urinary bladder cell proliferation. Toxicol Appl Pharmacol.

[B52] Aragones SN, Palacios DM, Avello dM, Gomez RP, Martinez CM, Rodriguez Bernabeu MJ (2001). [Arsenic levels in drinking water supplies from underground sources in the community of Madrid]. Rev Esp Salud Publica.

[B53] Smith AH, Lopipero PA, Bates MN, Steinmaus CM (2002). Public health. Arsenic epidemiology and drinking water standards. Science.

[B54] ATDSR (2000). Public Health Statement for Arsenic CAS# 7440-38-2. http://www.atsdr.cdc.gov/toxprofiles/phs2.html.

[B55] Edlich RF, Winters KL, Long WB (2005). Treated wood preservatives linked to aquatic damage, human illness, and death – a societal problem. J Long Term Eff Med Implants.

[B56] Belluck DA, Benjamin SL, Baveye P, Sampson J, Johnson B (2003). Widespread arsenic contamination of soils in residential areas and public spaces: an emerging regulatory or medical crisis?. Int J Toxicol.

